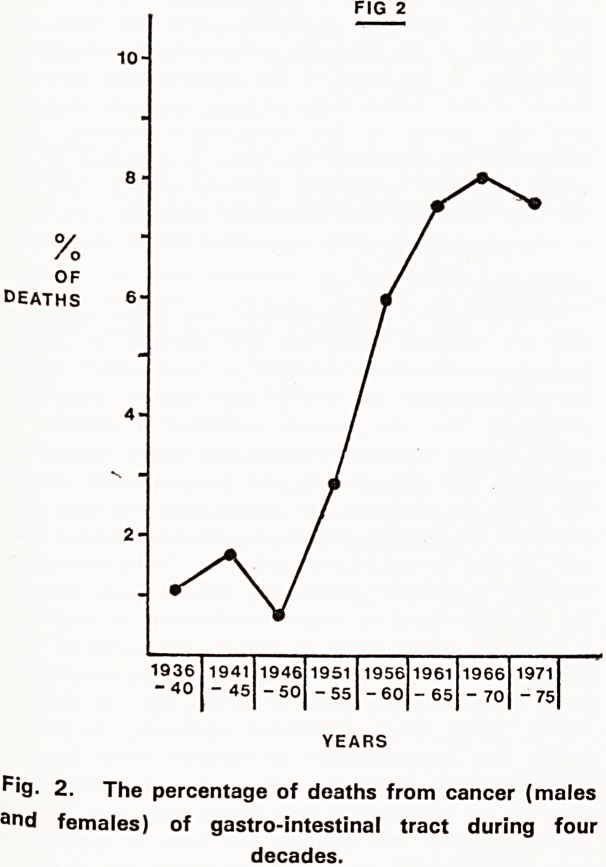# Cancer and Mental Retardation

**Published:** 1977

**Authors:** M. P. Jancar, J. Jancar

**Affiliations:** Stoke Park Hospital Group, Bristol; Stoke Park Hospital Group, Bristol


*Bristol Medico-Chirurgical Journal. Vol. 92. No. 341/342*

### Cancer and Mental Retardation

(A Forty Year Review)*

by

**M. P. JANCAR**

and

**J. JANCAR, MB, BCh, BAO, FRCPsych, DPM**

Stoke Park Hospital Group, Bristol

**Introduction**

When studying the records of deceased patients, while preparing a paper on "Twin Studies" (Jancar, 1970) it was noticed that the percentage of deaths from cancer had increased during the past two decades (1956-1975) compared with the two previous decades (1936-1955) (Fig. 1).

As a result of this observation, and being unable to find much information concerning deaths from cancer in mentally retarded patients, we decided to examine all the records of the patients who died from cancer during the past forty years in the Stoke Park Group of Hospitals in Bristol. We selected the forty year period for the following reasons.

(a) We wanted to compare the incidence of cancer before and after the introduction of antibiotics and tranquillizers during the 50's.

(b) During this period the hospital population has remained fairly constant at around 1,690 patients of which 820 were males and 870 females, equivalent to 32,800 and 34,800 patients respectively over the 40 year period.

(c) Records and detailed post mortem examination were started in 1933 by the late Professor Berry and Neuropathologist, Dr. Norman (Jancar, 1969) and since then post mortems have been performed, on average, on 48 per cent of the patients who have died in the Hospital Group. Some cases of cancer were diagnosed in vivo.

**Fig. 1. The percentage of deaths for all types of cancer (males and females) during four decades.**

**Population**

The Stoke Park Group of Hospitals caters for the mentally retarded of both sexes and all ages. The patients in the four hospitals were admitted from 44 counties of England and Wales. The total number of deaths during the period 1936-1975 was 1,125 (536 males and 589 females) of whom 81 (7.2 per cent) (30 males and 51 females) died from cancer. This indicates a higher percentage of death from cancer in females (8.7 per cent) than in males (5.6 per cent).

These figures when related to the population at risk translate into Death Rates/1000 Stoke Park population:

| | | | | |
|---|---|---|---|---|
| All causes | 16.3 | males | 16.9 | females |
| Cancer deaths | 0.91 | males | 1.47 | females |

These rates may be contrasted with National Data at the midpoints of each of our 20 year periods:

***Read at the Fourth International Congress of the International Association for the Scientific Study of Mental Deficiency Washington, DC, USA (August, 1976).**

**Death rates per 1,000 population (England and Wales)**

| | All causes | | Cancer | | Cancer as % all causes | |
|---|---|---|---|---|---|---|
| | M | F | M | F | M | F |
| 1947 | 13.6 | 11.3 | 2.10 | 1.89 | 17% | 17% |
| 1966 | 12.4 | 11.1 | 2.53 | 2.03 | 21% | 18% |

Unlike the population at large, the women in Stoke Park have higher crude death rates than men, both in total and from cancer in particular. However, although the hospital population experiences higher death rates than the country as a whole, cancer rates are low and cancer rates form a much lower proportion of all deaths.

**Diagnostic details**

The following table illustrates the grouping of various types of cancer in the 81 patients.

| Site of Cancer | | Male | Female |
|---|---|---|---|
| 1. **Gastro-intestinal tract** | (47) | | |
| Tongue | | — | 1 |
| Soft Palate | | 1 | — |
| Oesophagus | | 2 | 8 |
| Stomach | | 12 | 11 |
| Head of Pancreas | | — | 2 |
| Colon | | 1 | 4 |
| Rectum | | 4 | 1 |
| 2. **Genito-urinary tract** | (10) | | |
| Ovary | | — | 2 |
| Fallopian Tube | | — | 1 |
| Uterus | | — | 3 |
| Vulva | | — | 1 |
| Kidney | | 2 | — |
| Testis | | 1 | — |
| 3. **Respiratory system** | (3) | | |
| Bronchus | | — | 1 |
| Lung | | 2 | — |
| 4. **Breast** | (13) | — | 13 |
| 5. **Others** | (8) | | |
| Brain | | — | 1 |
| Bone | | — | 2 |
| Cauda Equina | | 1 | — |
| Leukaemia | | 2 | — |
| Lymphosarcoma | | 1 | — |
| Tonsil | | 1 | — |
| **Total** | **81** | **30** | **51** |

As compared with more general populations (Registrar General's Statistical Report: Milnes Walker 1972) this analysis reveals high proportions of cancer of the stomach in the male patients (40 per cent), followed by cancer of the rectum (13.3 per cent) in our special population. In the female patients the highest incidence is cancer of the breast (25.5 per cent,) closely followed by cancer of the stomach (21.6 per cent). The incidence of cancer of the oesophagus is also quite high in the females (15.7 per cent) whilst the other types of cancer were less frequently observed.

**Proportionate distribution of cancers**

| Males: Stoke Park % | Males: (1967) England & Wales % | | Females: Stoke Park % | Females: (1967) England & Wales % |
|---|---|---|---|---|
| 7 | 3 | Oesophagus | 16 | 2 |
| 40 | 13 | Stomach | 22 | 11 |
| 17 | 13 | Rectum & colon | 10 | 16 |
| — | — | Breast | 26 | 20 |

This relatively high proportion of upper alimentary cancers within a low cancer death rate overall is due to the fact that, although the conclusion is based on small numbers, cancer of oesophagus and stomach appear in the hospital population at or above community rates:

| | **Males** | | **Females** | |
|---|---|---|---|---|
| | **Stoke Park** | **England & Wales** | **Stoke Park** | **England & Wales** |
| Oesophagus | .06 | .07 | .23 | .05 |
| Stomach | .37 | .32 | .32 | .22 |

It is interesting to note that none of the 115 patients suffering from Down's syndrome who died during these forty years had cancer. However, there was one male patient with partial features of Down's syndrome who died from cancer of the stomach. At Stoke Park death patterns 8 cancer deaths would be expected in a series of 115 deaths and at England and Wales pattern 24.

This finding highlights the problem of drawing conclusions based on data which has not been standardized in respect of its age structure. The hospital population as a whole experiences higher death rates and earlier deaths than does the community from other causes, but within the hospital population mongols die earlier than average. This represents a considerable number of life years lost, and a reduced likelihood of surviving long enough to develop cancer.

FIG 2

**Fig. 2. The percentage of deaths from cancer (males and females) of gastro-intestinal tract during four decades.**

The average age at death for the population as a whole was about 69 years in 1966 whilst in Subnormality Hospitals it has been quoted at 40 for males and 42 for females. Only 5 per cent of cancers occur in the under 40's and approximately 50 per cent of cancer is seen in the 40-69 age range.

Applying national 1966 age/sex specific cancer death rates to the Stoke Park population at that time suggests that over 40 years such a unit would expect 99 cancer deaths. The figure of 81 observed cases does not differ significantly from this, and indeed the figure of 99 may well be a high standard in that the population at risk (especially the males) would have been younger in the earlier decades.

If the numbers, then, are roughly as expected the diagnostic mix is not. Carcinoma of the stomach has medium ages of onset and death later than those for cancer generally and may thus be regarded as a cancer of older populations.

**Age on admission and lengths of stay**

The age of the patients varied from 4 to 56 years. On average the male patients were admitted at the age of 19½ and females at the age of 24 years. The patients length of stay ranged from under a year to 62 years. On average the male patients stayed 37 years while the female patients 32 years. The males spent 63.6 per cent and the females 55.8 per cent of their life in the hospitals.

**Age at death**

The average age at which both male and female patients suffering from cancer died was 56½ years. The average age of death increased noticeably through the period of forty years. The average age during the last five years (1971-75) being double the average age during the first five years (1936-40).

**Tranquillizers**

29.6 per cent of the patients were on tranquillizers. Of the 30 males with cancer 9 were on tranquillizers. Of the 51 female patients 15 were on tranquillizers. The average duration of administration of tranquillizers was 6.6 years. The most commonly used tranquillizer was Largactil (15 cases), then Reserpine (10 cases) and less commonly used were Fentazin, Stelazine, Serenace, Neulactil and Melleril. In some cases more than one tranquillizer was used. There was no apparent relationship between the use of various tranquillizers and any one site of cancer.

**Psychotic Episodes**

One male patient and four female patients suffered from superimposed psychotic episodes.

**Family History of Mental Disorders**

35 per cent of the patients (11 male and 17 female) had relatives who suffered from mental disorders.

**Epilepsy**

9.9 per cent of the patients who died from cancer (3 male and 5 female) were epileptic.

**The patients' IQ's and Mental ages**

The IQ's of the patients who died from cancer ranged from 14 to 104. The average IQ for the females was 43 which was insignificantly higher than the average for the males which was 41. The patient's mental ages ranged from below 2 to 14.8 years. The average mental age for the females was 6.6 years and for the males 6.2 years.

**Discussion**

When examining our findings we noted that the life expectancy had increased during the past forty years, and particularly over the past twenty years after the introduction of antibiotics and better care of patients, as has been confirmed by various studies in England (Heaton-Ward, 1968, Richards and Sylvester, 1969) Sweden (Forssman and Akesson, 1970) the United States (Tarjan et al, 1969) and the recent Canadian survey (Balakrishnan and Wolf, 1976).

It is possible that some cases of cancer passed undetected, 58 per cent of cases of cancer were found in the gastro-intestinal tract (Fig. 2). The site most frequently affected was the stomach (12 males and 11 females) which occurred in over one third of all the cases. Seven of these patients (four males and three females) were receiving tranquillizers. The next most frequently affected site was the breast (13 females). Five of these were receiving tranquillizers including two who were on reserpine, which has been suspected of being carcinogenic through previous studies involving data from the Bristol Cancer Registry (Armstrong, et al, 1974). Six out of the eight epileptics who died from cancer had cancer of the gastro-intestinal tract and were all on anti-convulsant therapy.

At the National Cancer Institute and the American Cancer Society Conference in Florida in 1974, a number of papers on cancer aetiology and control were read. Reserpine was discussed among other drugs and was stated as a risk factor in breast cancer and possibly other tumours in persons treated for hypertension (Hoover and Fraumeni, 1975). In our survey there were ten patients on reserpine (five male and five female). Two females on reserpine had breast cancer, two had cancer of the stomach and one cancer of the head of the pancreas. Of the males, three had cancer of the stomach and two cancer of the rectum.

The third most frequent site was the oesophagus 10 cases (2 male and 8 female), which represent 12.3 per cent of the total. Of these one male and three females were receiving tranquillizers.

While comparing the figures for deaths from cancer in our patients with those for the general population in the South Western region of the United Kingdom in which our hospitals are situated, it was noted that the incidence rates were very much lower in the hospital than in the outside population. When comparing the average age of cancer deaths of the hospital patients and the general population it was found that male patients in hospital died five years earlier than those in the general population whilst the female hospital deaths occurred on average ten years younger than in females dying from cancer in the rest of the region (Dent, 1976). Another interesting observation is that 5 patients suffered from superimposed psychotic episodes, and 25 patients who died from cancer had familial history of mental disorder.

In our survey, seven patients (three males and four females) had signs of a clinical syndrome. Of the males, one with cancer of the rectum had Renpenning Syndrome, another with cancer of the cauda equina had muscular dystrophy, and the third who had cancer of the stomach suffered from encephalitis lethargica. In the case of the females, one who had cancer of the rectum suffered from psoriasis. Another, who had cancer of the oesophagus, suffered from congential syphillis and one who had cancer of the breast, suffered congenital absence of the right kidney and diabetes. Iron deficiency anaemia was noted in eleven patients (four male and seven female).

**Conclusion**

In conclusion, in our survey, we were unable to find any definite cause for the increase in cancer, and gastro-intestinal cancer in particular, among our patients or to apply any known theory of causation, but the survey revealed a number of points which might be helpful for future search and re-search into the causes and prevention of cancer. We wish to conclude our paper with the last paragraph of the leading article — "Macrophages v Cancer" in the Lancet of last July — "These war-games theories of tumour growth are intellectually pleasing but perhaps have as much relationship to reality as a game of chess does to human life patterns. This brings us back to the starting point, that people who think about tumours have to oversimplfy in order to derive some kind of modus operandi. As Burnet points out, sophistication in research often has had little practical application. The research-worker operates on the assumption that sweet logic will triumph in the long-run; the clinician will have to continue to base his activities on hypotheses which are obviously only part of the truth. In the next few years it will probably become clear how big a part of the truth are the contemporary notions of a natural and immunological defence mechanism against tumours. Whether to stimulate the phagocytes is still part of a doctor's dilemma."

**Acknowledgement**

We wish to thank Dr. N. A. Dent, Regional Specialist in Community Medicine (Information and Research), Bristol, for providing the national and regional figures of incidence of cancer and for the help with statistical analysis; to Mrs. P. Watts for the help in searching for the relevant patient's records and to Mrs. C. Ryan and Mrs. M. Fenton for the secretarial work.

### REFERENCES

Anon. (1976). Macrophages v Cancer. Lancet **2**, 27.

Armstrong, B., Stevens, N. and Doll, R. (1974). Retrospective Study of the Association between Use of Rauwolfia Derivatives and Breast Cancer in English Women. Lancet **2**, 672.

Balakrishnan, T. R. and Wolf, L. C. (1976). Life Expectancy of Mentally Retarded Persons in Canadian Institutions. Amer.J.ment.Defic. **80** No. 6,650.

Dent, N. A. (1976). Personal Communication.

Forssman, H. and Akesson, H. O. (1970). Mortality of the Mentally Deficient: A Study of 12,903 Institutionalized Subjects. J.ment.Defic. Res., **14**, 276.

Heaton-Ward, W. A. (1968). The Life Expectation of Mentally Subnormal Patients in Hospital. Brit. J. Psychiat., **114**, 1591.

Hoover, R., and Fraumeni, J. F. (1975). Drugs p. 189 in — Persons at High Risk of Cancer. An Approach to Cancer Etiology and Control. Fraumeni, J. F. Ed., Academic Press. inc. (London) Ltd.

Jancar, J. (1969). Sixty years of Stoke Park Hospital (1909-1969). Bristol Med-Chir. J. **84**, 77.

Jancar, J. (1970). Twins with Mental Retardation and Physical Abnormalities (Preliminary Report). Acta-Genet. Med. Gemellol, **19**, 311.

Milnes Walker, R. (1972). Cancer in South West England. A Review of Incidence and Survival for the Years 1955-1969 (South Western Regional Hospital Board).

Registrar General's Annual Statistical Reviews of England and Wales, Part 1, Table 7. H.M.S.O.

Richards, B. W., and Sylvester, P. E. (1969). Mortality Trends in Mental Deficiency Institutions. J.ment.Defic. Res. **13**, 276.

Tarjan, G., Eyman, R. K. and Miller, C. R. (1969). Natural History of Mental Retardation in a State Hospital Revisited. American Journal of Disturbed Children **117**, 609.

## Figures and Tables

**Fig. 1. f1:**
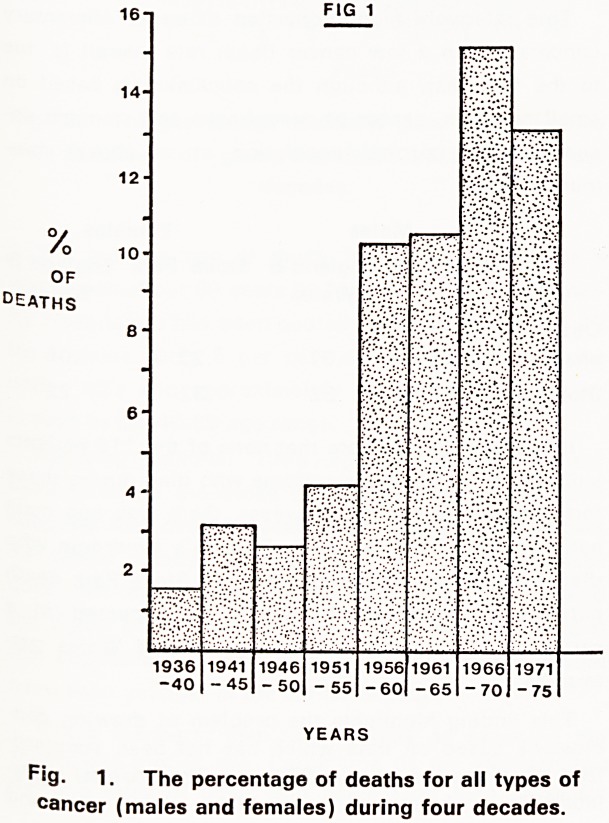


**Fig. 2. f2:**